# Effectiveness of curettage and bone grafting with and without elastic intramedullary nailing in the treatment of simple bone cyst in children: a meta-analysis

**DOI:** 10.3389/fsurg.2025.1633136

**Published:** 2025-10-21

**Authors:** Siyu Li, Lintao Wang, Yanan Li, Shuai Li, Dan Wang, Zhen Dong

**Affiliations:** 1 School of Clinical Medicine, Shandong Second Medical University, Weifang, Shandong, China; 2 Pediatric Orthopedics, Women and Children's Hospital Affiliated to Qingdao University, Qingdao, Shandong, China

**Keywords:** simple bone cyst/unicameral bone cyst (SBC/UBC), curettage and bone grafting, elastic intramedullary nailing, meta-analysis, children

## Abstract

**Aim:**

To systematically evaluate the effectiveness of curettage and bone grafting with and without elastic intramedullary nailing in the treatment of simple bone cyst in children by analyzing domestic and international literature.

**Methods:**

Systematically search for literature comparing the efficacy of curettage and bone grafting with and without elastic intramedullary nailing in the treatment of simple bone cyst in children in databases such as Pubmed, Cochrane Library, Embase, CBM, CNKI, Wanfang, and VIP. Conduct quality assessment and data extraction of included literature and perform meta-analysis calculations using Stata12.0 software.

**Results:**

Meta-analysis showed no heterogeneity (*P* = 0.581, I² = 0%). The cure rate was significantly higher in the EIN group (OR = 2.66, 95% CI = 1.48–4.77, *P* = 0.001) and 81% lower refracture incidence (OR = 0.19, 95% CI = 0.04–0.87, *P* = 0.032) vs. curettage alone.

**Conclusion:**

Curettage and bone grafting with elastic intramedullary nailing is superior to curettage and bone grafting alone for treating simple bone cysts, with lower postoperative refracture incidence. Although the latter may require only one surgery theoretically, refracture or recurrence could necessitate 2–3 additional surgeries.

## 1 Introduction

Simple bone cyst is a common benign tumor-like lesion in children, accounting for about 3% of all bone tumors (1). It originates from the medullary cavity, characterized by hidden and expansive growth, interfering with normal bone formation, reducing bone strength, and inducing pathological fractures (2). Simple bone cysts can occur in any skeletal site, especially the proximal femur and humerus, and spontaneously regress in 15% of cases (3), though active lesions require intervention. Pathogenesis remains unclear, with trauma (4), local venous obstruction (5), and genetic factors (6) proposed as related factors. The mainstream theory is local venous obstruction, leading to a lack of unified treatment. Current common treatments include curettage combined with autologous or allogeneic bone grafting, first proposed by Neer et al. in 1970. Other treatments include hormone or bone marrow injection, lesion excision and bone grafting, and arthroscopic treatment (7).

Elastic stable intramedullary nailing (EIN) is easy to operate and causes minimal trauma. For long bone cysts, EIN can continuously drain cyst fluid, reduce damage to bone, promote fluid circulation between the marrow cavity and lesion, destroy the cyst wall, lower local pressure, improve venous flow, and stabilize fractures. However, after EIN implantation, some patients do not achieve satisfactory outcomes. Based on these characteristics, domestic scholars applied EIN combined with focal curettage and mixed bone grafting to long bone cysts, achieving good results. Yet, EIN may damage the metaphysis, affect bone growth, and risk displacement or infection. Without EIN, curettage and bone grafting alone increases postoperative pathological fracture risk. Whether EIN combined with curettage and bone grafting avoids these issues and is more effective lacks evidence-based reports. This study uses evidence-based methods to evaluate the effectiveness of curettage and bone grafting with vs. without EIN for pediatric simple bone cysts, providing data for clinical decision-making.

## 2 Materials and methods

### 2.1 Inclusion and exclusion criteria

#### 2.1.1 Types of studies included

Randomized controlled trials, prospective studies, or retrospective cohort studies.

#### 2.1.2 Study subjects inclusion criteria

Patients with simple bone cyst under age 18, regardless of region, race, gender, lesion site, or size. Study designs: RCT, prospective, or retrospective cohort studies comparing curettage and bone grafting with vs. without EIN. Prognostic evaluation uses modified Capanna (8) and MSTS (9) scoring standards. Exclusion: recurrent SBC, prior treatment, other genetic metabolic diseases; case reports without controls.

#### 2.1.3 Interventions

Treatment of simple bone cyst with or without EIN combined with curettage and bone grafting.

#### 2.1.4 Outcome measures

Cure status, complications, recurrence per literature standards.

### 2.2 Literature search

**Figure 1:**
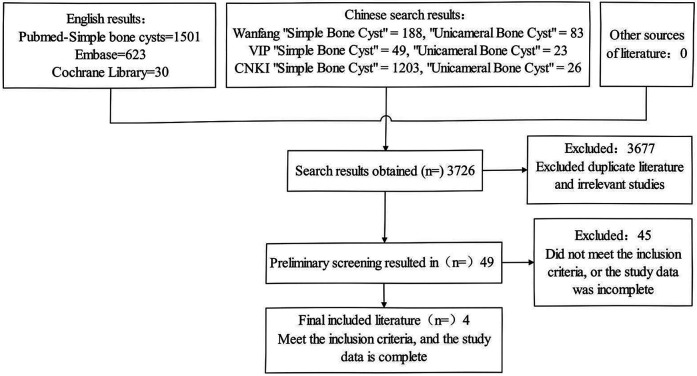
Literature search process and results.

Search Pubmed, Cochrane Library, Embase for “Simple bone cyst,” “Unicameral bone cyst,” “Solitary bone cyst.” Full-text search in CNKI, topic search in Wanfang, title + keyword search in VIP, up to November 2024. Include Chinese and English literature. Two authors assessed and screened literature. Screening process shown in (Figure 1).

### 2.3 Quality assessment

Included literature represented by first author and year. Evaluate based on study design, subject inclusion, characteristics, and imaging. Assess bias risk of retrospective studies using NOS criteria (9 points total; ≥7 high quality, <7 low quality) (10).

### 2.4 Data extraction

Two researchers independently screened literature per inclusion/exclusion criteria, completed quality assessment and data extraction. Disagreements resolved by a third reviewer. Extract title, first author, year, study type, age range, mean age, gender, follow-up, outcome standards. Parameters predicting surgical success (age, gender) extracted for correlation analysis. Per Capanna and MSTS, complete and basic healing considered success; partial and non-healing as failure.

### 2.5 Statistical processing

Meta-analysis using Stata12.0. Categorical data as frequency; binary variables as odds ratio (OR) with 95% confidence interval (95% CI). Homogeneity tested with Q-test. I² < 50%: fixed-effect model; I² ≥ 50%: analyze heterogeneity sources. If no significant clinical heterogeneity and no specific statistical source, use random-effects model. α = v0.05.

## 3 Results

### 3.1 Test results

Keyword searches initially identified 3,726 articles. After removing duplicates and irrelevant studies via title/abstract screening, 49 articles remained. After full-text review, 4 studies included: 3 English, 1 Chinese, totaling 254 cases (EIN group: 127, non-EIN group: 127). Basic characteristics and methodological quality (NOS) shown in Table 1.

**Table 1:** The characteristics of inclusion literature.

Study	Study type	Study region	Ethnicity	Sample size	Treatment		Age (years)		Follow-up (months)	Outcome variable
					EIN	NEIN	EIN	NEIN		
Erol (11)	Retrospective study	Istanbul	Caucasian	37	16	21	14 (7–17)	10 (3–17)	26–85	①②③④⑤⑦⑧
Zhang (12)	Retrospective study	China	Asian	62	30	32	8.1 ± 2.7	8.3 ± 3.4	12–93	①②③④⑤⑥⑦
Wang (13)	Retrospective study	China	Asian	48	25	23	10.8 ± 1.0 (7–16)	10.9 ± 1.1 (7–17)	8–16（11.3 ± 1.2）	①③④
Tang (14)	Retrospective study	China	Asian	107	56	51	9.62 ± 2.19	9.96 ± 2.27	26	①②③④⑤⑦⑧

EIN: curettage and mixed bone grafting with elastic intramedullary nailing; NEIN: curettage and mixed bone grafting without elastic intramedullary nailing; Outcome variable: ① cured; ② partial healed; ③ ineffective rate; ④ effective rate; ⑤ healing time; ⑥ infection; ⑦ recurrent fracture; ⑧ recurrence.

Notably, included studies provided limited data on cyst anatomical location and activity status (active vs. latent). Erol 2017, Wang 2021, and Tang 2022 cases were all humeral; only Erol 2017 and Zhang 2019 described activity. Lack of data precluded subgroup analyses on combined technique superiority in aggressive or specific lesions.

### 3.2 Quality assessment of included literature

All four studies were retrospective cohorts. Per NOS, three scored >7 (high quality), one (Erol 2017) scored <7 (low quality) (Table 2).

**Table 2:** Methodological quality evaluation of retrospective cohort study.

Included studies	Selection of study population	Comparability between groups	Outcome measurement	Total score
Erol (11)	3	2	1	6
Zhang (12)	4	2	3	9
Wang (13)	3	2	3	8
Tang (14)	3	2	2	7

### 3.3 Meta-analysis results

#### 3.3.1 Comparison of efficacy

**Figure 2:**
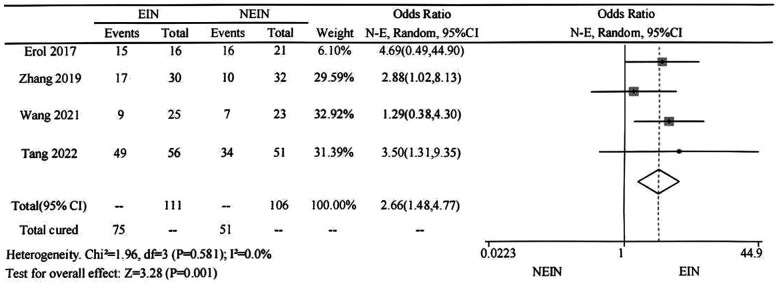
Meta-analysis of the efficacy of EIN and NEIN in the treatment of children with SBC: comparison of cure.

In the four included studies, the cure rates of the two methods were reported in detail, with 127 children receiving curettage and bone grafting with elastic intramedullary nailing and 127 receiving curettage and bone grafting alone. Meta-analysis indicated that there was no heterogeneity among the groups (*P* = 0.581, I² = 0.0%), and a fixed-effect model was used. The forest plot shows that the cure rate of curettage and bone grafting with elastic intramedullary nailing is higher than that of curettage and bone grafting alone in the treatment of simple bone cyst in children, and the difference is statistically significant (OR = 2.66, 95% CI = 1.48–4.77, *P* = 0.001), indicating that curettage and bone grafting with elastic intramedullary nailing is superior to curettage and bone grafting alone in the treatment of simple bone cyst in children (Figure 2).

#### 3.3.2 Comparison of postoperative refracture

**Figure 3:**
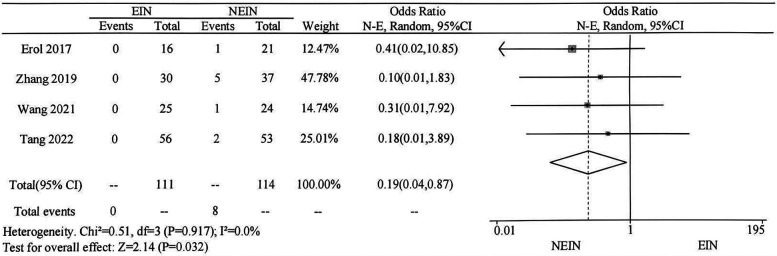
Meta-analysis of the efficacy of EIN and NEIN in the treatment of children with SBC: comparison of postoperative refracture.

In the four included studies, the occurrence of refracture after treatment was described. Meta-analysis indicated that there was no significant heterogeneity among the groups (*P* = 0.917, I² = 0.0%), and a fixed-effect model was used. The forest plot shows that there is a statistical difference in the number of postoperative refracture cases between the two groups (OR = 0.19, 95%CI = 0.04–0.87, *P* = 0.032), indicating that curettage and bone grafting alone is more likely to result in refracture after treatment of simple bone cyst in children (Figure 3).

#### 3.3.3 Comparison of gender and age

**Figure 4:**
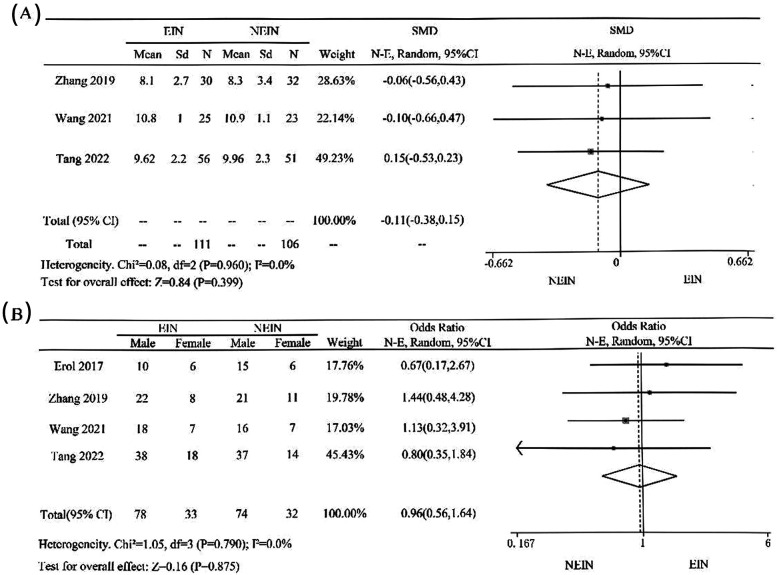
Meta-analysis of the efficacy of EIN and NEIN in the treatment of children with SBC: Comparison of age **(A)** and gender **(B)**.

In the four included studies, Zhang 2019, Wang 2021, and Tang 2022 detailed the age of children in the groups. Meta-analysis indicated that there was no significant heterogeneity among the groups (*P* = 0.960, I² = 0.0%), and a fixed-effect model was used. The forest plot (Figure 4A) shows that there is no statistical difference in age between the two groups (OR = −0.11, 95% CI = −0.38–0.15, *P* = 0.399); all four studies described the gender of children in the groups, with a total of 177 males and 77 females. Meta-analysis indicated that there was no significant heterogeneity among the groups (*P* = 0.790, I² = 0.0%), and a fixed-effect model was used. The forest plot (Figure 4B) shows that there is no statistical difference in gender between the two groups (OR = 0.96, 95% CI = 0.56–1.64, *P* = 0.875), indicating that the gender and age of children in the two groups have no significant effect on the efficacy.

#### 3.3.4 Sensitivity and bias analysis

**Figure 5:**
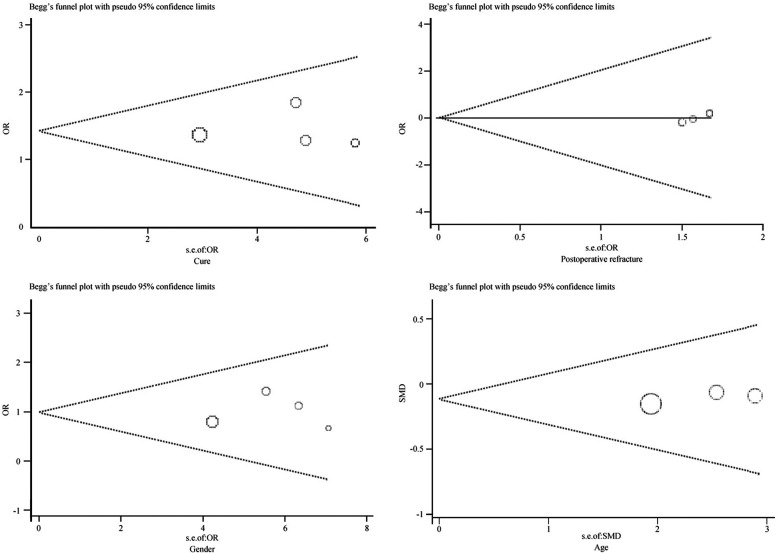
Sensitivity analysis.

**Figure 6:**
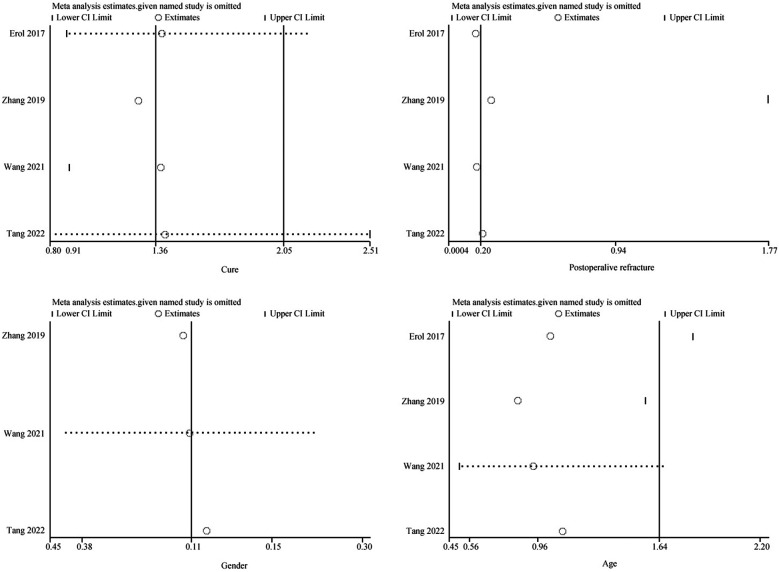
Publication bias.

Sensitivity and bias analysis was performed for cure, refracture, age, and gender, the stability was observed by one-by-one elimination method. The results show that after changing the effect size model, the results remained consistent, indicating that our conclusions are robust under different models. (Figure 5) In bias analysis, the Egger test *P* = 0.891 for cure, Begg test *P* = 0.308; Egger test *P* = 0.043 for refracture indicated potential small-study bias, Begg test *P* = 0.089; Egger test *P* = 0.380 for age, Begg test *P* = 1.000; Egger test *P* = 0.832 for gender, Begg test *P* = 1.000, and the funnel plots show symmetry, indicating that these results have no significant publication bias (Figure 6).

## 4 Discussion

This meta-analysis provides evidence that the combination of curettage and bone grafting (CBG) with elastic intramedullary nailing (EIN) yields superior outcomes compared to CBG alone for pediatric simple bone cysts (SBC), specifically in terms of higher radiographic cure rates and a significantly reduced risk of postoperative refracture. This study represents the first meta-analysis to quantify the synergistic effect of EIN augmentation in pediatric SBC treatment, overcoming limitations of single-center studies and providing higher-level evidence for clinical decision-making.

While the combination of open curettage, bone grafting, and nailing represents a specific surgical strategy, its clinical value is particularly evident in addressing the primary weakness of standalone curettage: mechanical instability during the healing phase. This combined approach is especially relevant for cases involving pathological fractures at presentation or large, aggressive cysts in weight-bearing bones with a high imminent fracture risk. The EIN provides immediate internal stabilization, protecting the grafted cavity from collapse and facilitating earlier mobilization, while the curettage and grafting aim to eliminate the cyst biologically. Our findings quantify this potential synergistic effect.

As a benign self-limiting disease, children with simple bone cysts often seek medical attention due to pathological fractures, and most require surgical treatment to alleviate pain at the site, restore the function of the affected limb, improve the quality of life of children, and improve prognosis. Donaldson and others’ research (11) indicates that some patients with bone cyst can heal on their own, but local lesions cannot heal. Open curettage and bone grafting and bone grafting is the traditional surgical method, which has a large trauma and a high incidence of postoperative refracture. Combining with elastic intramedullary nailing can effectively reduce the recurrence rate, and the surgical method of curettage and bone grafting and bone grafting combined with elastic intramedullary nailing only requires two surgeries, reducing the fear of repeated hospitalization for children, and is one of the main methods for treating simple bone cyst at present.

Curettage and bone grafting and autologous or allogeneic bone grafting, first proposed by Neer and others in 1970, according to Wang and Han (12) and Flont and others’ research (13), the cure rate is different when using and not using EIN in focal curettage and bone grafting and bone grafting. According to Mavčič and others’ research (1), the recurrence rate with EIN is lower than with drainage screws. In the four included literatures of this study, the cure rates of the two treatment methods were reported in detail, and the meta-analysis suggests that curettage and bone grafting with elastic intramedullary nailing has a higher cure rate than curettage and bone grafting alone, and the incidence of postoperative refracture is low, which is consistent with the results of other scholars (12, 14–16).

The 81% reduction in refracture incidence with EIN augmentation is a critical finding. This translates to a substantial clinical benefit by potentially avoiding the need for 2–3 repeat surgeries per 10 patients, addressing a key endpoint concerning reoperation rates.

However, for cyst near the epiphyseal end, using this surgical method may penetrate the cortical bone, damage the epiphyseal plate and surrounding nerves and other soft tissues. In addition, the tail of EIN may stimulate subcutaneous tissues, leading to local inflammation, hyperplasia, and ulcers. If, in order to avoid damage to the epiphyseal plate, EIN is implanted too shallowly, it will affect its support and drainage function, leading to unsatisfactory treatment effects (1). However, the proximal end of the EIN may irritate subcutaneous tissues, causing local inflammation and ulcers (17). In the four included literatures, only Wang described the recurrence after surgery, in addition, Wang also described the Neer and VAS scores of shoulder joint function before and after surgery, showing that the EIN group is better than the NEIN group, and the existing evidence still cannot prove the postoperative recurrence of the two treatment methods.

Elastic intramedullary nails have the effect of fixing the diaphysis to prevent the occurrence of fractures, so this study compared the occurrence of refracture after surgery. In the included literatures, all four described the occurrence of postoperative refracture, and the meta-analysis suggests that the incidence of refracture in the EIN group is lower than in the NEIN group. EIN-augmented curettage reduced refracture by 81% vs. curettage alone-clinically avoiding-repeat surgeries per 10 patients.

This study extensively searched domestic and foreign literature, covering multiple countries and regions, and finally included four retrospective studies, which were quality-evaluated using the NOS evaluation criteria. This is the first meta-analysis quantifying EIN's synergistic effect, overcoming single-center limitations. Three of them were high-quality studies, and one (Erol 2017) was considered a low-quality study due to the lack of detailed description of the results. The limitations of this study are: (1) The source of the implanted bone is different, graft heterogeneity (autologous/allogeneic ± marrow) may confound healing: Tang used allogeneic bone powder and combined it with autologous bone marrow injection, and the other three did not describe the source of the bone graft material, nor did they use other treatment methods. (2) The follow-up years: The measurement years of the outcome indicators in the included literatures vary, Erol and Tang have a follow-up year of more than two years, Zhang has a minimum follow-up year of one year and a maximum of more than 7 years, Wang's follow-up year is within two years, with an average of 11.3 years, and the difference in follow-up time may affect the accuracy of recurrence cases and cure cases, causing bias to the results. (3) A significant limitation of this analysis is the lack of detailed data on cyst location and activity status in the primary studies. This prevented us from performing subgroup analyses to determine if the combined technique is particularly advantageous for more aggressive lesions (e.g., active cysts, specific anatomical locations), which would be a valuable indicator for its use. (4) In the treatment, factors such as lesion volume, cavity partitioning, preoperative pathological fracture situation, lesion activity, lesion site, etc., may affect the efficacy; the literature only partially reported the above-mentioned influencing factors, among which Zhang reported the preoperative fracture situation and lesion volume, Wang reported the preoperative fracture situation, and there is a certain dispute, so the included literature in this study could not analyze the related factors. (5) The common complications after the two surgical methods are pathological fractures, and the rare complications are pain, infection, local discomfort, hardware irritation in some EIN cases, etc. Furthermore, important endpoints such as physeal injury and growth disturbance were not sufficiently reported in the included literature, thus precluding related analysis. (6) Both surgical methods are open surgery, and only Zhang and Wang described the operation time and blood loss, and the gap is large, so no analysis comparison was made. Secondly, since only Chinese and English literature were retrieved and included, there may still be publication bias.

In summary, EIN combined with CBG may reduce refracture risk and improve cure rates compared to CBG alone, though evidence on recurrence and long-term complications remains limited. Due to the limitations of publication bias and the number of included cases, there are certain limitations, so more large-sample, high-quality RCTs are needed to further confirm the results of this paper. Future studies should prioritize the systematic collection and reporting of data on cyst location, activity status, and long-term growth-related outcomes to better define the optimal indications for this combined procedure. This research contributes to the field by providing quantitative evidence for the synergistic effect of EIN augmentation and identifying key areas for future investigation to optimize surgical management of pediatric simple bone cysts.

## Data Availability

The raw data supporting the conclusions of this article will be made available by the authors, without undue reservation.
